# EFRAILTY.ORG: ANALYSIS OF INITIAL USERS

**DOI:** 10.1093/geroni/igae098.3963

**Published:** 2024-12-31

**Authors:** Megan Cheslock, Stephanie Sison, Lily Zhong, Natalie Newmeyer, Kuan-Yuan (Ken) Wang, Andrea Schwartz, Ariela Orkaby, Dae Hyun Kim

**Affiliations:** Veterans Affairs-Bedford (Edith Nourse Rogers Memorial Veterans Hospital), Bedford, Massachusetts, United States; University of Massachusetts Chan Medical School, Worcester, Massachusetts, United States; University of Connecticut School of Medicine, Farmington, Connecticut, United States; Hebrew SeniorLife, Boston, Massachusetts, United States; Hebrew SeniorLife, Boston, Massachusetts, United States; Harvard Medical School, Boston, Massachusetts, United States; Brigham and Women’s Hospital, Boston, Massachusetts, United States; Hebrew SeniorLife, Boston, Massachusetts, United States

## Abstract

In March 2024, eFrailty.org was launched to the public with the goal of making frailty assessment tools accessible to clinicians and researchers. We report the uptake and initial user experiences of eFrailty between January 1, 2024 and July 31, 2024, as measured by Google Analytics. eFrailty was viewed over 23,000 times by more than 5,400 users. More than 1000 users (18%) returned after their initial visit. There were 4 distinct spikes in usage coinciding with the airing of a GeriPal Podcast episode, two international geriatrics conferences, and publication of a NEJM review article—all of which mentioned eFrailty. Analytics data indicated the site was accessed from over 60 different countries. The highest number of users were from the United States (2,400 users), followed by China (802), and Canada (522). The most frequently visited site feature was the ‘Help Me Choose a Frailty Tool’ page, which guides the user through a series of questions to identify an assessment tool that is best aligned to their purpose and available information. Of the 15 validated frailty assessment tools featured, the three most frequently viewed were the CGA-FI (2,105 views), Clinical Frailty Scale (709), and FRAIL Scale (680). User feedback was gathered informally from local and international colleagues using the site, and via e-mails sent through the site’s ‘Contact Us’ link. In response to feedback about the CGA-FI, the authors created a dedicated instruction manual. These initial data suggest the utility of eFrailty website for clinical care, research, and education.

